# Self-care concept for people with elimination ostomy: a scoping review

**DOI:** 10.1590/1980-220X-REEUSP-2024-0041en

**Published:** 2024-12-20

**Authors:** Cristiane Rabelo Lisboa, Josimare Aparecida Otoni Spira, Eline Lima Borges

**Affiliations:** 1 Universidade Federal de Minas Gerais, Escola de Enfermagem, Belo Horizonte, MG, Brazil.

**Keywords:** Colostomy, Ileostomy, Ostomy, Self Care, Rehabilitation, Colostomía, Ileostomía, Estomía, Autocuidado, Rehabilitación, Colostomia, Ileostomia, Estomia, Autocuidado, Reabilitação

## Abstract

**Objective:**

To analyze the literature for terminology, classifications, and factors influencing the adoption of self-care in people with an elimination ostomy.

**Method:**

Scoping review, according to JBI methodology and, for structuring the article, the extension of the PRISMA checklist. The search included studies from 2018 to 2023, in four databases, with specific descriptors and alternative terms. Two reviewers selected the sample based on inclusion/exclusion criteria, using the software Rayyan^®^. The protocol was registered in the *Open Science Framework*.

**Results:**

Eight studies were included, which presented differences in the scope of the concept and classification of self-care. Names such as capacity, index, and level were used. Four tools were identified for assessing self-care. The factors that positively influenced self-care were female sex, being young, married, higher education level, pasty effluent, demarcation, social support, and education. Those influencing negatively were stress, complications, and inadequate care.

**Conclusion:**

The concept of self-care for people with elimination ostomies is not standardized and is most often reduced to procedural self-care.

## RESUMO

**Objetivo:**

Analisar na literatura as terminologias, classificações e fatores que influenciam a adoção do autocuidado em pessoas com estomia de eliminação.

**Método:**

Revisão de escopo, conforme metodologia JBI e, para a estruturação do artigo, a extensão do checklist PRISMA. A busca contemplou estudos de 2018 a 2023, em quatro bases de dados, com os descritores específicos e termos alternativos. Dois revisores selecionaram a amostra mediante critérios de inclusão/exclusão, usando o programa Rayyan^®^. O protocolo foi registrado na *Open Science Framework*.

**Resultados:**

Incluídos oito estudos, que apresentaram diferenças na amplitude do conceito e na classificação do autocuidado. Foram utilizadas denominações como capacidade, índice e nível. Quatro ferramentas foram identificadas para avaliação do autocuidado. Os fatores que influenciaram positivamente o autocuidado foram sexo feminino, ser jovem, casado, maior escolaridade, efluente pastoso, demarcação, suporte social e educação. Os que influenciaram negativamente foram estresse, complicações e assistência inadequada.

**Conclusão:**

O conceito de autocuidado das pessoas com estomias de eliminação não é padronizado, sendo que, na maioria das vezes, é reduzido ao autocuidado procedimental.

## RESUMEN

**Objetivo:**

Analizar en la literatura las terminologías, clasificaciones y factores que influyen en la adopción del autocuidado en personas con estoma de eliminación.

**Método:**

Revisión del alcance, según la metodología del JBI y, para estructurar el artículo, la ampliación del checklist PRISMA. La búsqueda incluyó estudios de 2018 a 2023, en cuatro bases de datos, con descriptores específicos y términos alternativos. Dos revisores seleccionaron la muestra según criterios de inclusión/exclusión, utilizando el programa Rayyan^®^. El protocolo fue registrado en *Open Science Framework*.

**Resultados:**

Se incluyeron ocho estudios que mostraron diferencias en la amplitud del concepto y clasificación del autocuidado. Se utilizaron designaciones como capacidad, índice y nivel. Se identificaron cuatro herramientas para evaluar el autocuidado. Los factores que influyeron positivamente en el autocuidado fueron el género femenino, ser joven, estar casado, educación superior, efluente pastoso, demarcación, apoyo social y educación. Los que influyeron negativamente fueron el estrés, las complicaciones y la asistencia inadecuada.

**Conclusión:**

EL concepto de autocuidado para personas con ostomías de eliminación no está estandarizado y, en la mayoría de los casos, se reduce a un autocuidado procedimental.

## INTRODUCTION

Amid the complexity of health care, self-care stands out as a primary need in promoting well-being and effectively managing chronic conditions. Understanding and facilitating self-care in specific populations, including those living with an elimination ostomy, is essential to care.

Elimination ostomies are highly frequent in the adult population, the most common being colostomy, ileostomy, and urostomy^(1)^. Annual estimates of surgical procedures for ostomy placement are approximately 120,000 in North America^(2)^, being between 100,000 and 130,000 in the United States^(3)^, and 13,000 in Canada^(4)^. The number of people living with an elimination ostomy is 650,000 to 730,000 in the United States^(5)^, 700,000 in Europe^(6,7)^, 150,000 in Germany^(8)^, 20,000 in Portugal^(9)^, and 207,000 in Brazil^(10)^.

The projection of the *International Ostomy Association* (IOA) considers that there is one person with an ostomy for every thousand inhabitants in countries with a good level of medical care, and it may be much lower in less developed countries^(10)^. Even though it is not accurate, the estimated number of people with an elimination ostomy confirms the need to seek knowledge on the subject, especially considering the challenges that these people face after the procedure.

Surgery, although life-saving, involves important changes in a person’s lifestyle and habits^(11)^. Bowel or urinary incontinence and, consequently, the need to use a collection device, can cause discomfort, embarrassment, and insecurity to the person. It is common to be concerned about exposure, rupture, and leakage when faced with the need for constant equipment emptying and cleaning^(12)^. People with an ostomy may experience psychological disorders in social and family relationships^(13)^, as well as face social stigma, with the potential to interfere with acceptance and adaptation to the new condition^(12)^.

The person with an ostomy must be supported to face the challenges arising from the new situation, regardless of whether the ostomy is temporary or permanent. To contribute to this process, aiming at rehabilitation, the development of self-care is essential. Self-care is a key concept for people with chronic illnesses, including those with ostomies^(14)^. The concept, widely adopted in the health field, refers to an individual’s ability to take care of themselves, promoting, maintaining, or improving their health. This definition is supported by the theory of self-care, which is a valuable tool for directing care and tends to improve the quality of nursing care provided^(15)^. In fact, it should be used to provide nurses with tools to assess people with an elimination ostomy.

The use of a specific self-care assessment tool can ensure the measurement of the outcomes achieved. For people with an ostomy, self-care is the desirable outcome. This means that self-care can be considered an important indicator of rehabilitation, as well as being useful for assessing the quality of care provided by services aimed at assisting these people.

Due to its complexity, it is considered that the theory of self-care requires appropriation of the content for its adoption in clinical practice in the care setting for people with ostomies. This fact raises the following question: what does scientific production have to say about the concept, classification, and factors related to self-care for people with elimination ostomy in the hospital and extra-hospital setting?

Having a clear answer to the question presented is important for decision-making when adopting interventions. Healthcare professionals have to consider the different aspects of the life of the person with an ostomy to identify areas where self-care skills are lacking. These actions can foster specific educational interventions to improve the impact that living with an ostomy has on people^(14)^.

The synthesis of knowledge on the topic will support the selection of the concept and classification of self-care to be adopted by nursing assistants. Furthermore, it will also contribute to the implementation of self-care strategies. This scoping review aims at analyzing the literature for terminology, classifications, and factors influencing the adoption of self-care in people with an elimination ostomy.

## METHOD

### Design of Study

This is a scoping review, which follows the model of the method *Joanna Briggs Institute* (JBI)^(16)^ and the guidelines of the *checklist Preferred Reporting Items for Systematic reviews and Meta-Analyses extension for Scoping Reviews* (PRISMA-ScR)^(17)^. The study protocol was previously prepared and made available in the repository *Open Science Framework* (OSF) with number DOI 1017605/OSF.IO/MU48S, accessed at: https://osf.io/mu48s/.

A scoping review follows a broad systematic approach to map scientific literature on a topic and to identify key concepts, theories, sources, and knowledge gaps^(18)^. This type of method was considered more appropriate to analyze the terminology, classifications, and factors influencing the adoption of self-care in people with an elimination ostomy.

It should be noted that scoping reviews map the existing evidence on a given subject without analyzing the methodological quality of the included studies, as their purpose is not to find the best evidence, but to define how it was produced and in what contexts. The study followed a methodological strategy consisting of six steps: 1) identification of the research question; 2) identification of relevant studies; 3) selection of studies; 4) data extraction; 5) separation, summarization, and reporting of results; and 6) presentation of results^(16)^.

### Identifying the Guiding Question

The guiding question for this review was formulated according to the PCC mnemonic^(18)^, considering 3 items: P – Population: person with elimination ostomy; C – Concept (central question to be examined): concept, classification, and factors related to self-care; C – Context: hospital and extra-hospital setting. Through PCC, this review guiding question was formulated: what does scientific production have to say about the concept, classification, and factors related to self-care for people with elimination ostomy in the hospital and extra-hospital setting?

### Sources of Information and Eligibility Criteria

Experimental and quasi-experimental study designs were considered in this review, including randomized and non-randomized clinical trials, before-and-after studies, and time series. Additionally, descriptive and analytical observational studies, such as prospective and retrospective cohort studies, case-control studies, cross-sectional studies, case series, and individual case reports, were included. Qualitative studies and literature reviews, consensuses and guidelines were also considered.

The inclusion of publications in English, Portuguese and Spanish, published between 2018 and 2023, was established. The language restriction was due to linguistic viability. Date restriction considered the evolution and current status of knowledge about self-care in ostomy. Eligible study participants were adults or older people, regardless of sex and duration of intestinal ostomy. The identified studies would have to address a set of or at least one of the variables: concept, scope, classification, facilitating and hindering factors of self-care. Studies carried out both in hospital and extra-hospital settings were eligible.

Duplicate studies and those that had adolescents, children, or newborns with an elimination ostomy as participants or that involved the caregiver of people with an ostomy were excluded. Abstracts and conference proceedings, notes, reports, reviews, and letters were also excluded.

### Search Strategy

The search strategy was developed based on terms extracted from the research question. Controlled descriptors were identified in the Health Sciences Descriptors (DeCS)/Medical Subject Headings (MeSH) via the Virtual Health Library (VHL). The descriptors were combined using the Boolean operators OR and AND. The search was conducted in May 2023 and included three stages. The first stage was to search for articles in the VHL metabase, as well as in the Scopus, Web of Science, and MEDLINE databases (access via PubMed). The second stage considered publications classified as grey literature (organization websites). Finally, a search was carried out in the references of the articles in the bibliographic sample (search for citations).

The final strategy for information retrieval, including all descriptors and related terms for each database, was defined according to the access base. A total of 267 publications were identified in VHL, 490 in MEDLINE/PubMed, 47 in Scopus, and 17 in Web of Science.

- VHL: (estomia OR ostomia OR ostomy OR ostomies OR colostomia OR colostomies OR colostomy OR ileostomia OR ileostomy OR ileostomies) AND (autocuidado OR autoajuda OR “self care” OR “self-care” OR “care, self” OR “self help” OR autogestão OR autogerenciamento OR “auto gestão” OR “auto-gerenciamento” OR “auto-gestão” OR autogerenciamento OR “self-management” OR automanejo) AND (“estado funcional” OR “functional status” OR “functional dependence” OR “functional independence” OR “functional status” OR reabilitação OR rehabilitation OR rehabilitación OR habilitação).
- MEDLINE/PubMed: (ostomy OR ostomies OR colostomies OR colostomy OR ileostomy OR ileostomies) AND (“self care” OR “self-care” OR “care, self” OR “self help” OR “self-management”) AND (“functional status” OR “status, functional” OR “independence, functional” OR “functional dependence” OR “functional independence” OR “functional status” OR rehabilitation).
- Scopus:(ostomy OR ostomies OR colostomies OR colostomy OR ileostomy OR ileostomies) AND (“self care” OR “self-care” OR “care, self” OR “self help” OR “self-management”) AND (“functional status” OR “status, functional” OR “independence, functional” OR “functional dependence” OR “functional independence” OR “functional status” OR rehabilitation).
- Web of Science: (ostomy OR ostomies OR colostomies OR colostomy OR ileostomy OR ileostomies) AND (“self care” OR “self-care” OR “care, self” OR “self help” OR “self-management”) AND (“functional status” OR “status, functional” OR “independence, functional” OR “functional dependence” OR “functional independence” OR “functional status” OR rehabilitation).

### Study Selection

The studies found in the VHL and in the MEDLINE/PubMed, Scopus, and Web of Science databases were exported to the software Rayyan® for reference management and duplicate removal. Two nurses, both with a master’s degree, one of them a stomatherapist and the other a researcher on the subject, independently conducted the screening of titles and abstracts, choosing the studies for full reading. Disagreements in the selection were resolved by consensus between two judges, one with a master’s degree and the other with a doctorate degree, both specialists in stomatherapy. Following the selection, data were extracted.

### Data Extraction and Analysis

Data extraction and analysis stage involved a thorough assessment by three reviewers, all ostomy care nurses, two with a master’s degree and one with a doctorate degree. To extract and organize the data, the reviewers prepared a form in Microsoft Excel® 2019, containing data such as article title, year of publication, country, language, objectives, study design, population, sample, scenario, and level of evidence. In addition, a second form was created to organize the main results, including data on self-care: concept, scope, classification, and influencing factors. A pilot test was conducted to verify reviewers’ understanding and ensure consistency of the approach taken in the study selection process. It was not necessary to make any adjustments to the forms.

Two of these reviewers worked together to separate, summarize, and report the essential elements of each study. Articles were coded numerically in ascending order to facilitate reference (E1 to E8). Any disagreement was resolved through discussion with the third reviewer, who played the role of judge in the previous phase. This collaborative approach ensured consistency in data extraction and analysis.

Following JBI protocol^(16)^, data were grouped to reflect the main or recurring themes related to self-care for people with an elimination stoma and the factors that influence its adoption. The included studies were analyzed to identify the terminologies used in the context of self-care, concepts, domains and dimensions, classifications and main factors that positively or negatively influence its adoption. The outcomes are presented in a table with the selected studies characterization. The first figure describes the process of identifying and selecting studies. The second figure brings together the concept of self-care organized according to its scope. The scope of self-care (capacity, index and level) and tools used for its assessment, finding similarities between studies, are presented in the third figure. The summary of the factors that influence the adoption of self-care, in terms of ease and difficulty, is described in the fourth figure.

### Ethical Aspects

As this is a review study with public domain data, without the involvement of human beings, the assessment of the Research Ethics Committee was waived.

## RESULTS

**Figure 1:**
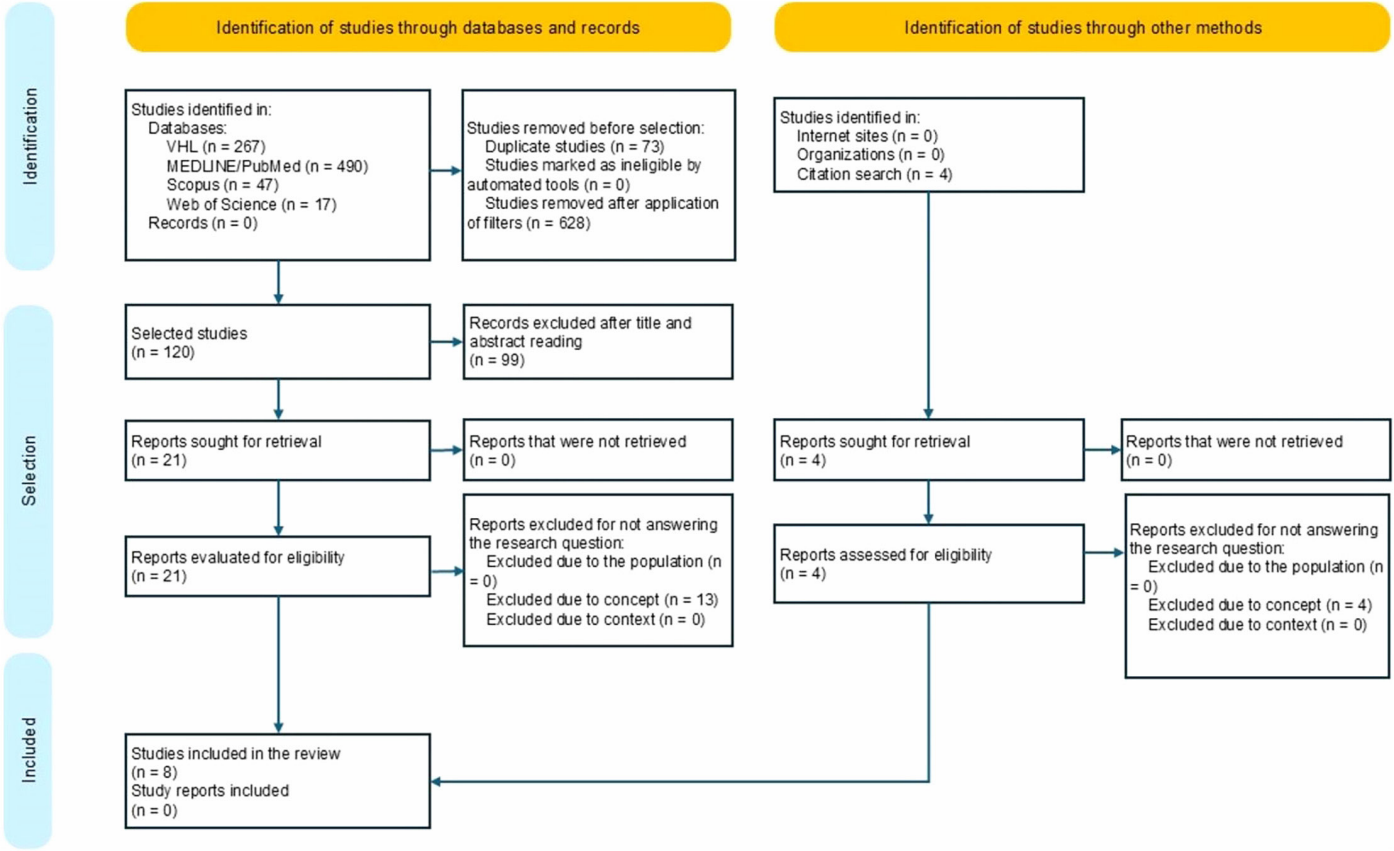
PRISMA flowchart of the selection of review articles. Belo Horizonte, MG, Brazil, 2023.

Source: Prepared by the authors based on PRISMA 2020.

A total of 821 publications were retrieved from the databases. With the application of the eligibility criteria and removal of duplicates, a total of 120 articles were obtained. After reading titles and abstracts, both evaluators selected 21 articles. After reading the selected articles, one evaluator selected ten articles and the other selected six, with only two being coincident. The analysis of the divergences by the third evaluator resulted in the composition of the sample with eight articles, seven of which were found in MEDLINE/PubMed and one in VHL. Four reports were found through citation searching and were excluded because they did not answer the research question (Figure 1).

The selected articles (Chart 1) were published in English and produced in China (3), Spain (2), Italy (1), Brazil (1), and Australia (1), in the years 2018 (2), 2020 (2) and 2021 (4), three of which were carried out in a hospital setting. There was a predominance of studies with a cross-sectional design (3), followed by prospective (2), methodological (1), qualitative studies (1), and non-randomized controlled clinical trial (1).

**Chart 1:** Characterization of selected studies, Belo Horizonte, MG, Brazil, 2023.

Code/Reference	Objective	Design of study	Sample/population	Local
E1^(19)^	To investigate the effect of the FOCUS-PDCA procedure on the self-care ability of people undergoing colostomy due to rectal cancer.	Non-randomized controlled clinical trial	160 people with rectal cancer undergoing colostomy.	Out-of-hospital
E2^(6)^	To describe self-care in people with ostomies, identify sociodemographic aspects and clinical variables associated with self-care, and identify the association between self-efficacy for self-care and self-care beyond the variables associated with self-care.	Longitudinal, prospective, and multicentric	523 people with an ostomy (T0*) and 362 were followed up after 6 months (T1**).	Out-of-hospital
E3^(20)^	To describe the development of the Specific Self-Care Questionnaire for Ostomized Patients (CAESPO) instrument and to evaluate its construct validity, internal reliability, and test-retest reliability (temporal stability).	Methodological	125 people with temporary or permanent intestinal ostomy.	Out-of-hospital
E4^(21)^	To evaluate the correlation between intimate marital relationship, self-disclosure, and adaptability in people with colorectal cancer (ICC) with enterostomy.	Cross-sectional	390 people with ostomies due to colorectal cancer.	Out-of-hospital
E5^(22)^	To examine the relationship between perceived social support and self-care ability among Chinese people with enterostomy and explore whether perceived stress mediated this relationship.	Cross-sectional	410 people with intestinal ostomy, with 392 valid questionnaires.	Hospital
E6^(23)^	Interpret the self-care experience of people with enterostomy registered in an ostomy program, based on the framework of the Social Model of Disability.	Qualitative exploratory	9 people with intestinal ostomy	Out-of-hospital
E7^(24)^	To examine ostomy self-care and health-related quality of life in people with intestinal ostomy, describe clinical and sociodemographic variables and analyze the relationship between all of them.	Multicenter cross-sectional	139 participants with intestinal ostomy	Hospital
E8^(25)^	To determine the number of stoma therapist visits and the total time spent providing face-to-face care and education time required, on average, to achieve independence with an ostomy.	Prospective cohort	107 people hospitalized for colorectal surgery underwent ostomy formation.	Hospital

Note: *T0: initial time of the study.

**T1: six months after the start of the study.

**Figure 2:**
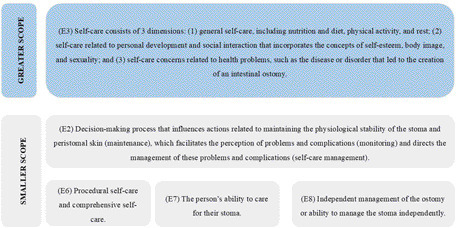
Self-care concepts presented by the sample. Belo Horizonte, MG, Brazil, 2023.

Studies E2, E3, E6, E7 and E8 presented the concept of self-care (Figure 2).

Five studies addressed the concept of self-care, one of which was considered to be broader, since the concept included knowledge, skills, and attitudes aimed at self-care performance. The remaining four were defined as having a smaller scope because the concept included procedural actions directed at the ostomy.

**Figure 3:**
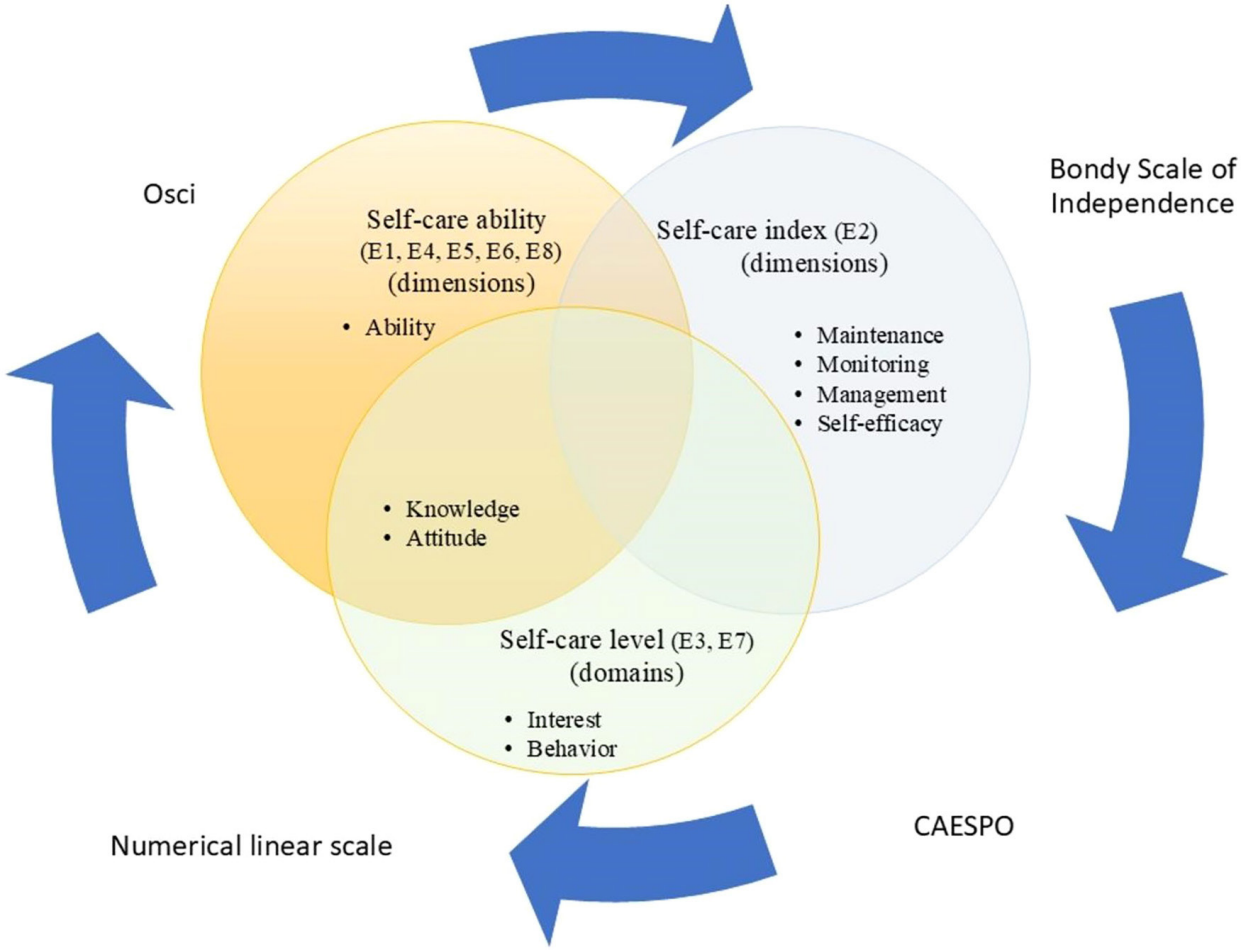
Scope of self-care and tools used for its assessment. Belo Horizonte, MG, Brazil, 2023.

The authors of the studies used different terms to characterize the scope of self-care: capacity, index and level, which in turn included dimensions or domains. Self-care was also classified using tools (Figure 3).

In studies in which self-care capacity was assessed, the authors used different tools, such as OSCI, CAESPO, Bondy Independence Scale, and the numerical scale. The assessment identified the capacity and index of self-care through dimensions, and the level through domains. The similarity between these two assessments was knowledge and attitude, present in studies E1, E3, E4, E5, E6, E7 and E8. In study E2, the self-care index was assessed considering the dimensions of maintenance, monitoring, management and self-efficacy.

**Figure 4:**
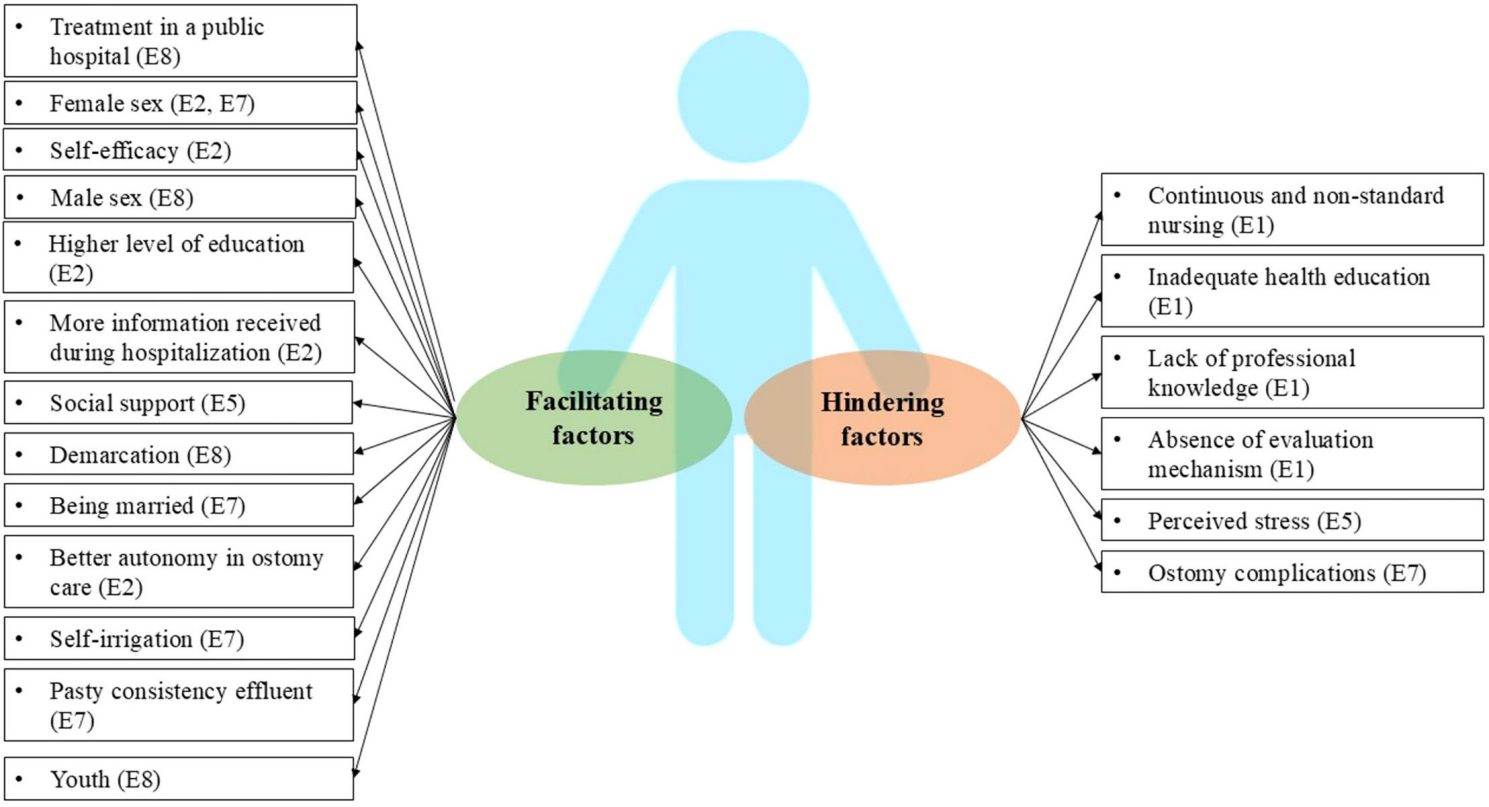
Factors influencing self-care of people with intestinal ostomy. Belo Horizonte, MG, Brazil, 2023.

Factors related to the individual with an ostomy, as well as health institutions (considering structure and process), influence the development of self-care (Figure 4).

Thirteen factors were identified as facilitators for the adoption of self-care, with females being cited in two articles and males in one article. In the group of hindering factors, six were identified and most of them were related to the actions of professionals and the institutional process.

Thus, of the eight studies that made up the sample, the concept of self-care was explained in five of them, with four focusing on procedural actions for the ostomy. The authors used different tools to assess self-care, classifying it into capacity, index and level. Nineteen factors that influenced the adoption of self-care were identified. Of these, 13 were facilitators and six were hindrances to the adoption of self-care.

## DISCUSSION

The result of this review showed that self-care for people with an elimination ostomy is an important aspect of care, since it is closely related to rehabilitation and quality of life. Although there are many studies on people with ostomies, there is still a shortage of studies published in the last five years on the subject, especially on the concept and classification of self-care.

The analysis of the identified self-care concepts reveals a striking divergence, highlighting the incipient understanding of this phenomenon in the context of people with ostomies. The concept of self-care varied considerably across studies. This fact signals gaps in the understanding of the scope of the concept of self-care. Of the five studies that presented the concept of self-care, three restricted it to specific care of the ostomy^(23, 24, 25)^. Therefore, the phenomenon had its scope reduced to the procedural aspect.

How an individual engages in self-care varies due to cultural and environmental influences^(15)^. The concept of self-care should not be restricted to caring for the ostomy and the collection equipment. This understanding of restricted self-care suggests a reductionist view of the phenomenon. Comprehensive care must be sought, covering all aspects of life, allowing for rehabilitation and quality of life^(23)^. Thus, procedural self-care is fundamental, but other aspects have to be considered.

In a broader sense, the concept of self-care encompassed the decision-making process, involving maintenance, monitoring, and management of complications^(6)^. There was progress in the comprehension of the concept when three dimensions were proposed: general self-care (nutrition, diet, physical activity, and rest), self-care related to personal development and social interaction (self-esteem, body image and sexuality) and self-care concerns related to health problems, such as the disease or disorder that led to the creation of the ostomy^(20)^.

Concepts of self-care have not advanced sufficiently to facilitate the rehabilitation of people with ostomies in an unequal and non-equitable society. Therefore, the concepts are not aligned with the fundamental assumptions of Dorothea Orem’s self-care theory^(15)^, which includes three interconnected theories, making it complex. In the group of theories mentioned, there is the theory of self-care, which describes why and how human beings take care of themselves. The other theory, self-care deficit, clarifies why and how human beings can be helped through nursing care. Finally, nursing systems theory describes the relationships between nurses and patients and the importance of these relationships for quality nursing care^(15)^.

People with an ostomy, based on article 5 of Decree No. 5.296, of December 2, 2004, were identified as “physically disabled” in Brazil. Considering their limitations and/or incapacities to perform activities, they thus began to have all the social protection granted to a person with disabilities in the legal system, at federal, state, and municipal levels^(26)^, obtaining the feature of an atypical person. People with ostomies face additional challenges in an inequitable society, which are made worse when self-care is reduced in scope. The existence and functioning of an ostomy are associated with changes in the person’s life that can affect their physical, social, emotional, and mental health^(5)^. This situation hinders this person’s ability to achieve a standard of living equivalent to that of a typical person (without a disability), further exacerbating existing inequalities.

When addressing the development of self-care, it was found that the authors of the review studies used different terms, such as self-care capacity, assessed through dimensions^(19, 21, 22, 23, 25)^, level of self-care, considering the domains^(20)^ and self-care index, classified by dimensions^(6)^. However, the common point for the development of self-care was restricted to the domain/dimension knowledge and attitude. This fact demonstrates the lack of standardization in the way self-care is assessed and confirms the difference in the scope of the concept.

According to the definition of the World Health Organization (WHO), self-care refers to the abilities of individuals, families and communities to promote health, prevent disease, maintain health and cope with illness or disability, with or without the support of a health professional^(27)^. Therefore, to assess the scope of self-care for people with an elimination ostomy, parameterizing its assessment is vitally important. Furthermore, this assessment can contribute to monitoring the quality of care in the health service.

The studies in this review addressed the classification of self-care through the use of different tools. Among these, OSCI^(6)^, CAESPO^(20)^, the Bondy Independence Scale^(25)^, and the revised Self-Care Assessment Scale for People with Chronic Conditions^(23)^. A study classified self-care into fully or partially capable of self-care, and unable to take care of oneself^(21)^. Another study^(23)^ classified the capacity for ostomy-specific self-care and management of the collection equipment as full, partial, and absent capacity. Despite these findings and the availability of some tools for assessing self-care, there is still a lack of consensus on the best classification for clinical practice and how to assess the progression of the scope of self-care by the person with a stoma.

For people to adapt to changes related to ostomy care and daily life, the priority is to support them in learning about ostomy management care^(28)^. This implies the need for integrated and planned education for self-care as a way of supporting these people in overcoming physical, psychological, and social difficulties^(29)^. At this stage of the rehabilitation process, caregivers can play an important role in the self-care process. This fact was confirmed by the multicenter study carried out in seven outpatient clinics in two Italian regions, involving 252 caregivers. The contribution of these caregivers was measured through the *Caregiver Contribution to Ostomy Self-Care Index* (CC-OSCI). However, it was found that caregivers with less education and those who lived with the person with an ostomy were less likely to contribute to self-care monitoring (perception of problems and complications). Spouse caregivers and those with greater preparation contributed significantly less to self-care management. The data suggest that there is a variety of sociodemographic factors associated with the caregiver’s contribution to the self-care of people with an ostomy^(30)^.

The use of different tools hinders language standardization and the comparison of results within services, including those with similar characteristics. Considering that self-care can be a marker or indicator for evaluating results, this review confirms the need for professionals to make efforts to reverse the identified situation.

The organization of the health network can impact the adoption of self-care by people with an ostomy since it is influenced by factors such as skills and experience, motivation, cultural beliefs and values, self-confidence, well-established habits, functional and cognitive skills, social support from other people, and access to care^(31)^. Access to educational actions for self-care and the development of autonomy in managing the ostomy are associated with greater physiological stability of the ostomy and peristomal skin, as well as greater perception of problems and complications^(31)^.

Health education must cover physical, psychological, and sociocultural aspects. It must be adapted to the reality and needs of people with an ostomy, as each person faces the condition in a unique way^(32)^. Health education facilitates changes in the way of thinking and acting. It is necessary for self-care, strengthening coping strategies and reducing the implications related to physical, psychological and social changes.

The factors considered positive for self-care were social support and being married. Family support is essential, especially at the beginning of the rehabilitation process. The family can motivate and contribute to the development of skills and abilities for ostomy care. Accordingly, it is important that the person feels accepted, embraced, and safe, which contributes to their adaptation to their new body image. Furthermore, self-esteem, autonomy, improved health status, and other emotional aspects influence the adoption of self-care^(32)^.

The presence of complications related to the stoma^(24)^, inadequate assistance^(19)^, and perceived stress^(22)^ were factors hindering self-care cited in the studies in this review. A survey conducted in the United States, with the aim of identifying the challenges related to collection equipment and self-care reported by cancer survivors, showed that bleeding, pain, effluent leakage, skin problems, allergic reactions to adhesives, problems related to the collection equipment, and the time required to care of the stoma were the factors most cited by participants^(33)^. Anxiety about leakage, odor, and skin irritation greatly inhibit social activity and self-confidence for people with ostomies.

The nurse has to understand the coping strategies of the person with an ostomy and work on their specific needs, considering their strengths and weaknesses to achieve autonomy^(34)^.

### Contributions to the Areas of Nursing and Health

The study allowed the identification of the divergences and limitations of the concept of self-care presented in various publications. The search for understanding the concept of self-care with an ostomy that goes beyond procedural care encouraged the authors of this review to propose the concept of self-care in people with an elimination ostomy: “self-care in people with an elimination ostomy refers to the actions and skills that these individuals develop to care for their ostomy independently. This includes practical aspects such as hygiene and changing collection equipment, as well as the ability to recognize problems, monitor ostomy health, and make informed decisions to manage complications, regardless of the setting in which these individuals are inserted. Self-care encompasses both the physical and emotional aspects of adapting to the condition of the person with an ostomy, aiming to improve quality of life and minimize complications.”

This proposition recognizes that self-care is not a uniform process, but rather a multifaceted practice that varies according to the individual experience, social context, and health conditions of the person with an ostomy. It respects the context of a theory: its explanation about people, nursing, health, and environment (metaparadigm). It considers self-care to be a practical endeavor because it includes explicit guidelines regarding human growth, development, and functioning. It is important to advance other studies that exemplify the role of nurses in self-care. In short, it is necessary to be clear about the “what”, “why”, “who” and “how” of self-care for people with elimination ostomy in what regards nursing practice.

Orem’s self-care theory can be used to develop assessment and guidance tools to measure the quality of care provided to people with an elimination ostomy. Therefore, it is important to be aware of the concept to be adopted for this specific audience.

The result of this review showed that self-care for people with an elimination ostomy is an important aspect of care for this public, since it is closely related to rehabilitation and quality of life. Although there are many studies on ostomy, this review demonstrated that there is a shortage of studies carried out and published in the last five years that address self-care, mainly clarifying its concept and classification.

Knowing the factors that interfere with self-care will enable nurses to carry out a more assertive assessment and implement specific interventions that can improve the self-care experiences of people with an ostomy. New studies in the area are encouraged, so that they can contribute to the standardization of concepts and classifications of self-care for a systematic evaluation of the person with an ostomy, applicable to the practice of care.

### Study Limitations

It should be noted that the classification of self-care presented in some studies in the sample was not accompanied by the instrument used for this purpose. This fact hampers the comprehension of the theoretical framework adopted by the authors of these studies, confirming the need to improve knowledge on this topic.

This scoping review presents limitations related to the method, such as the restriction to three languages, four databases, and the period from 2018 to 2023, which may have resulted in the exclusion of research published in other languages, indexed in other sources, and older studies that could answer the research question. Furthermore, theses and dissertations were not included, considering that these productions have their products published in the form of articles. Despite these limitations, it is considered that the objective of the study was achieved.

## CONCLUSION

The scoping review showed that the concept of self-care for people with elimination ostomies is not standardized and is most often reduced to procedural self-care. The literature does not assertively provide a classification of self-care that can be adopted in the practice of assisting people with elimination ostomies, making it difficult to define interventions and measure the desired results.

To contribute positively to the adoption of self-care, health education is a key factor, as well as being a woman, young, married, having a higher level of education, pasty effluent, demarcation, and social support. Stress, the presence of complications related to the stoma, and inadequate care are factors that hinder self-care and, consequently, the rehabilitation and quality of life of the person with an ostomy.

The concept and classification of self-care lack standardization. Carrying out further studies related to the topic, aiming at greater exploration and in-depth knowledge, could contribute to a better definition of the concept and classification of self-care, providing support for defining interventions and monitoring the results of the care provided.
